# Functional characterization of *Pseudomonas soli* VMAP1 as a biocontrol agent against *Xanthomonas vesicatoria* in tomato plants

**DOI:** 10.1038/s41598-026-45489-y

**Published:** 2026-03-30

**Authors:** Tadeo Elías Galván, Valeria Paola Conforte, João Carlos Setubal, Gabriela Petroselli, Rosa Erra-Balsells, Laila Toum, Natalia Mielnichuk, Florencia Malamud, Federico Coluccio Lescow, Florencia Denisse Navarro, Adrián Alberto Vojnov, Pablo Marcelo Yaryura, María Isabel Bianco

**Affiliations:** 1 Instituto de Ciencia y Tecnología Dr. César Milstein (Consejo Nacional de Investigaciones Científicas y Técnicas–Fundación Cassará), Buenos Aires, Argentina; 2 Instituto de Investigación en Medicina y Ciencias de La Salud, Universidad del Salvador, Buenos Aires, Argentina; 3 Departamento de Bioquímica, Instituto de Química, Universidade de São Paulo, São Paulo, Brazil; 4 Centro de Investigaciones en Hidratos de Carbono, Consejo Nacional de Investigaciones Científicas y Técnicas – Facultad de Ciencias Exactas y Naturales, Universidad de Buenos Aires (CIHIDECAR, CONICET-UBA), Buenos Aires, Argentina; 5 Departamento de Ciencias Básicas, Universidad Nacional de Luján, Buenos Aires, Argentina; 6 Instituto Multidisciplinario de Investigación y Transferencia Agroalimentario y Biotecnológica (IMITAB, UNVM-CONICET), Villa María, Córdoba Argentina; 7 Instituto Académico Pedagógico de Ciencias Básicas y Aplicadas, Universidad Nacional de Villa María, Villa María, Córdoba Argentina; 8 Instituto de Investigaciones en Ciencias Agrarias y Veterinarias, Universidad del Salvador, Buenos Aires, Argentina

**Keywords:** *Pseudomonas soli*, Biocontrol, Virulence attenuation, Plant defence, Outer membrane vesicles, *Xanthomonas vesicatoria*, Biotechnology, Microbiology, Plant sciences

## Abstract

Although several *Pseudomonas* spp. have recognised biocontrol properties, the ecological and functional potential of *Pseudomonas soli* remains largely unexplored. Here, the 5.6 Mb genome of the strain VMAP1 was sequenced and annotated to confirm its taxonomic identity and investigate genes associated with biocontrol traits. Genome mining and targeted in vitro and in planta assays were combined to assess its biocontrol potential. The genome harbours genes linked to the synthesis of bioactive compounds, as well as to regulatory and secretion systems homologous to those involved in biocontrol in other *Pseudomonas* spp. Functionally, VMAP1 exhibited motility and tolerance to changing environmental conditions. It also produced outer membrane vesicles and bioactive compounds including hydrogen cyanide, xantholysins and pseudopyronines. Diffusible VMAP1-derived metabolites impaired motility and biofilm formation in phytopathogen *Xanthomonas vesicatoria*, without inhibiting its growth. They also elicited plant defence responses, namely callose deposition and stomatal closure. Our results are consistent with earlier observations of a 75% reduction in the severity of bacterial spot in tomato plants treated with diffusible VMAP1-derived metabolites. They also offer novel insights into VMAP1’s ability to suppress the pathogen and induce host immune responses, and thus into its potential as a novel biocontrol agent for sustainable tomato production.

**Supplementary Information:**

The online version contains supplementary material available at 10.1038/s41598-026-45489-y.

## Introduction

Phytopathogens affect up to 40% of global food production and are thus responsible for major agricultural losses^1^. Current strategies for combating them include crop rotation, mechanical control, chemical pesticides, and the introduction of resistance genes into crops. However, in the face of environmental degradation and rising antimicrobial resistance, there is a growing demand for more eco-friendly management practices.

An alternative that is increasingly attracting interest is the use of biocontrol agents (e.g., living organisms or their metabolites) which are naturally able to fight plant disease. They may do so directly, through the production of metabolites that kill pathogens or inhibit their growth, or indirectly, by priming plant defence responses, detoxifying plants from harmful compounds, promoting plant growth, and enhancing plant tolerance to stress^2^.

Many bacteria with biocontrol properties inhabit the rhizosphere and belong to a group known as plant growth-promoting rhizobacteria (PGPRs)^3–5^. Among them, members of the genus *Pseudomonas* are particularly remarkable for their broad genetic and metabolic versatility, having relatively large genomes that make them adaptable to a wide variety of environments^6^. These bacteria synthesise an extensive repertoire of bioactive compounds that can suppress phytopathogens and/or stimulate plant growth^7^. Furthermore, they engage in sophisticated cell–cell signalling and can colonise diverse ecological niches through chemotaxis, adhesion and biofilm formation, in which key roles are played by flagella, type IV pili (T4P) and exopolysaccharides (EPSs)^7–9^. While considerable research has focused on their ability to suppress fungal pathogens, much less is known about their activity against bacterial phytopathogens. Besides, certain species are at present poorly characterised despite their promising biocontrol potential. This is the case of *Pseudomonas soli*.

Only a few *P. soli* strains have been studied since the species was first described in 2014^10^. Some of them have been reported to possess nematicide and antifungal properties, but their taxonomic identification was based exclusively on 16S ribosomal RNA gene sequencing^11,12^, an inadequate method for accurate species-level classification^9^. Their correct identification through genomic sequencing is the necessary first step towards a greater understanding of their metabolic, biocontrol, and/or plant growth-promoting capabilities.

To date, VMAP1 is the only *P. soli* strain for which biological activity has been experimentally validated in planta. This strain was isolated by our group from the rhizospheric soil of healthy tomato plants, and its biocontrol properties were demonstrated in the pathosystem *Xanthomonas vesicatoria* (*Xv*)–*Solanum lycopersicum* (tomato)^13^.

*Xv* is a phytopathogenic bacterium that causes bacterial spot in tomato and pepper (*Capsicum* spp.). In tomato, the disease is responsible for yield and fruit quality losses that can exceed 50% in highly vulnerable production systems, particularly under warm and humid conditions^14,15^. It is traditionally managed by rotating crops, planting less susceptible cultivars and applying copper-based agrochemicals^16^. Excessive use of these chemicals, however, poses environmental risks and contributes to the emergence of copper-resistant strains^17^. Once again, this underscores the urgent need for sustainable and effective control strategies.

The present study sought to build on our earlier findings by further examining the biocontrol potential of *P. soli* strain VMAP1 through a combination of genome mining and targeted phenotypic assays. The results obtained in silico, in vitro and in planta provide a robust framework for developing VMAP1-based formulations aimed at controlling bacterial spot in tomato in a sustainable manner.

## Results

### Genomic insights and in silico analysis of biocontrol-related traits

The VMAP1 genome comprises a 5.6 Mb circular chromosome with 64% GC content. Assembly yielded a single scaffold and six contigs, with ~ 800 × coverage depth. The Prokaryotic Genome Annotation Pipeline (PGAP) revealed 5,060 coding sequences (CDSs) and 80 RNA genes (Table S1). PLSDB and PLASMe did not detect any plasmids, which is consistent with the single-chromosome structure.

**Fig. 1:**
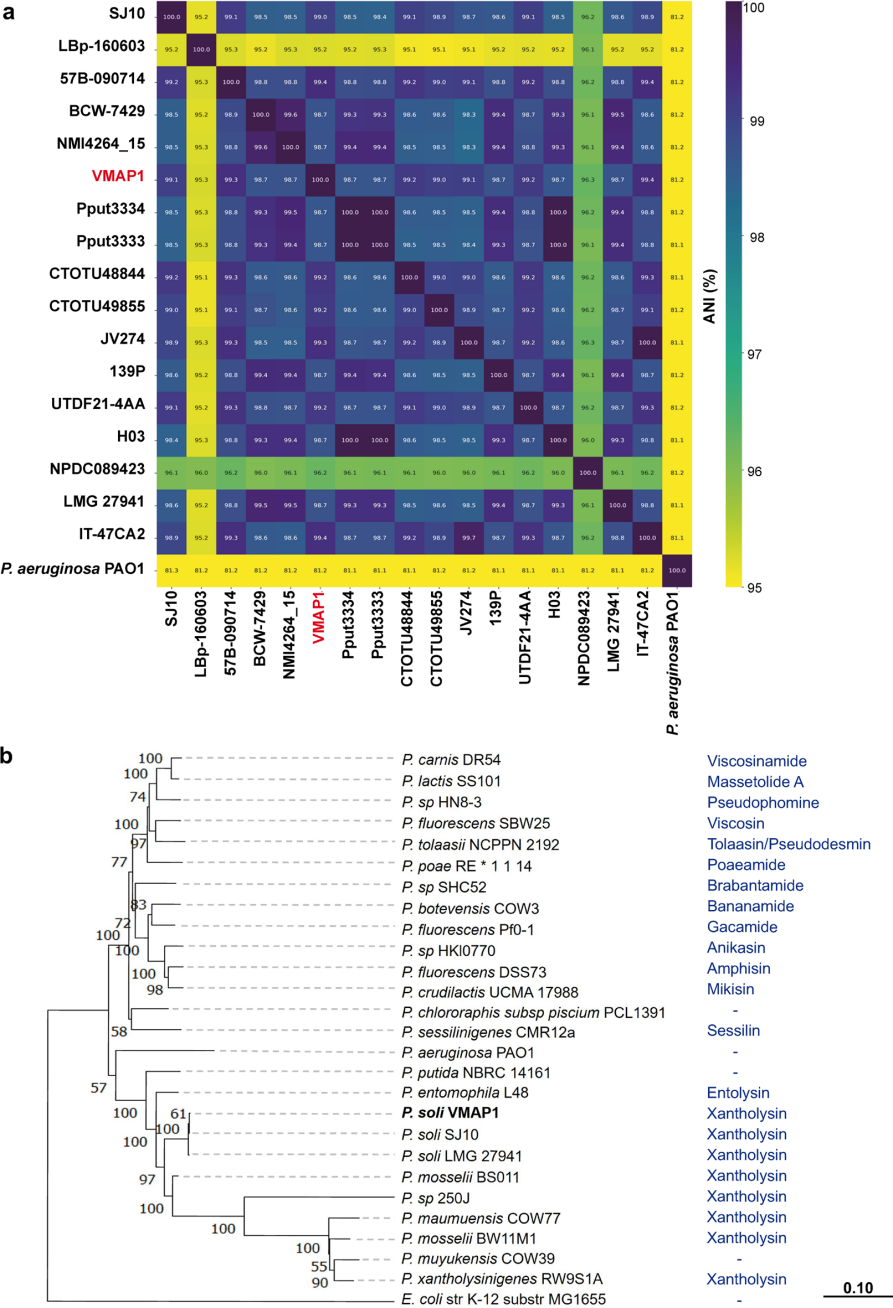
Taxonomic and phylogenetic analysis of *Pseudomonas soli* strain VMAP1. (**a**) Average Nucleotide Identity (ANI) between VMAP1 and *P. soli* genomes, with *Pseudomonas aeruginosa* PAO1 used as an outgroup. (**b**) Phylogenetic tree based on multilocus sequence analysis (MLSA) showing the taxonomic position of VMAP1 within the *Pseudomonas* genus. Bootstrap values (1,000 replicates) are shown at nodes. Predicted cyclic lipopeptides (CLPs) associated with each strain are indicated.

The average nucleotide identity (ANI) between VMAP1 and 16 other *P. soli* genomes was over 98%, which confirms that it is a member of the *P. soli* species (Fig. 1a). Its phylogenetic status was corroborated by a multilocus sequence analysis (MLSA) of concatenated gene fragments (*rpoD*^*346–1196*^*-pepN*^*1711–2571*^*-gltX*^*86–909*^), with 100% bootstrap support (Fig. 1b).

The largest COG (clusters of orthologous genes) categories were amino acid transport and metabolism, transcription, signal transduction mechanisms, translation ribosomal structure and biogenesis and cell wall/membrane/envelope biogenesis (Fig. S1). These are indicative of metabolic and adaptive versatility of VMAP1.

Some CDSs were homologous to gene clusters associated with motility and adhesion, including those involved in the biosynthesis of flagella, T4P and EPSs. Gene clusters for the production of alginate and the synthesis of *Pseudomonas* acid polysaccharide (Pap) were detected, whereas those encoding other EPSs commonly described in *Pseudomonas* spp. were absent (Table S2), which suggests the strain harbours a relatively limited repertoire of biofilm-associated EPSs.

The genome also contains genes encoding the GacS/GacA two-component regulatory system, alongside CDSs which are homologous to type VI secretion system (T6SS) genes described in closely related *Pseudomonas* spp. (*P. putida*, *P. mosselli* and *P. entomophila*). Sequence identity was high (≥ 80%) for CDSs homologous to *tssA, B, C, E, F, G, J, K, M, hcp* and *vgrG*, and lower (< 80%) for those homologous to *tssL* and *clpV1*. An incomplete type III secretion system (T3SS) cluster was identified, but there were no genes encoding PAAR-domain proteins. Some structural and regulatory gene sequences were highly identical (≥ 80%) with known *Pseudomonas* orthologues, while others were highly divergent or absent (Table S3).

Other CDSs were homologous to gene clusters for biocontrol-related compounds previously described in *Pseudomonas* spp. These include non-ribosomal peptide synthetase (NRPS) clusters for the production of xantholysins and pseudopyronines, as well as genes associated with the synthesis of hydrogen cyanide (HCN), pyrroloquinoline quinone (PQQ), a putative R-type pyocin and lytic enzymes (Table 1).

**Table 1:** Biocontrol-associated gene clusters and biosynthetic loci identified in the VMAP1 genome.

Most similar known cluster	Genes/gene cluster found in VMAP1 genome
Pseudopyronines A and B (PK-like)	*ppyS*
Xantholysin A, B and C (NRP-CLP)	*xtlABCDEFR*
HCN (VOC)	*hcnABC*
R-type pyocin (Bacteriocin. Phage tail-like)	*lexA, lydA, hol* (holin superfamily III), com (*fluMu-*like)*, pld1* (putative)*, gpV, gpW, baseplate gp*-like, *tail_gp, tssP* (putative)*, gpFII, tac1/tac2, gpU, gpX, rz, hyp1* (putative)*, hyp2* (putative)
PQQ (Redox co-factor)	*pqqABCDEF*
Proteases	*pfpI*, *impA*/*sppA*, *lasAB*, *U32p* (putative)*, piv, 57ara, paaP, putative protease,*
Peroxidases	*dyp, prx/bcp, tpx, katA, katB, katE, osmC, lsfA, ahpF, ahpB, ahpC*
Oxidases	*adi1, hemN, mnxG, mnxA/mcoA, cyoD, ccoQ, ccoN, ccoO, ccoP, maoA/aox* (putative)*, nfo, nox, nqr, nadB, pdxH*

Abbreviations: PK-like, polyketide-like (synthesised via PK-like pathways involving fatty acid synthase systems, which arenon-canonical PKS systems); NRP-CLP, non-ribosomal peptide–cyclic lipopeptide; VOC, volatile organic compound; PQQ,pyrroloquinoline quinone.

For other genes and gene clusters that were searched for in the VMAP1 genome but not detected, see Table S2.

### Phenotypic traits relevant to bacterial fitness and biocontrol

The growth dynamics of VMAP1 were studied under different conditions in vitro. In tryptone soy broth (TSB), it grew well at temperatures between 20 and 37 °C, tolerated 40 °C, but did not grow at 45 °C. The optimal temperature for growth was 28 °C, and was thus chosen for subsequent experiments. Adequate growth was likewise observed at pH values ranging from 6 to 10, with the optimal value being pH 7. VMAP1 was nevertheless able to survive at pH 4 and 12. When the medium was supplemented with NaCl to test tolerance to salinity, VMAP1 was able to withstand concentrations as high as 16%, but growth was better at concentrations of up to 8% and optimal at 0.5% NaCl. In addition, VMAP1 was resistant to ampicillin and chloramphenicol and sensitive to polymyxin B, tetracycline and aminoglycosides (Fig. S2).

**Fig. 2:**
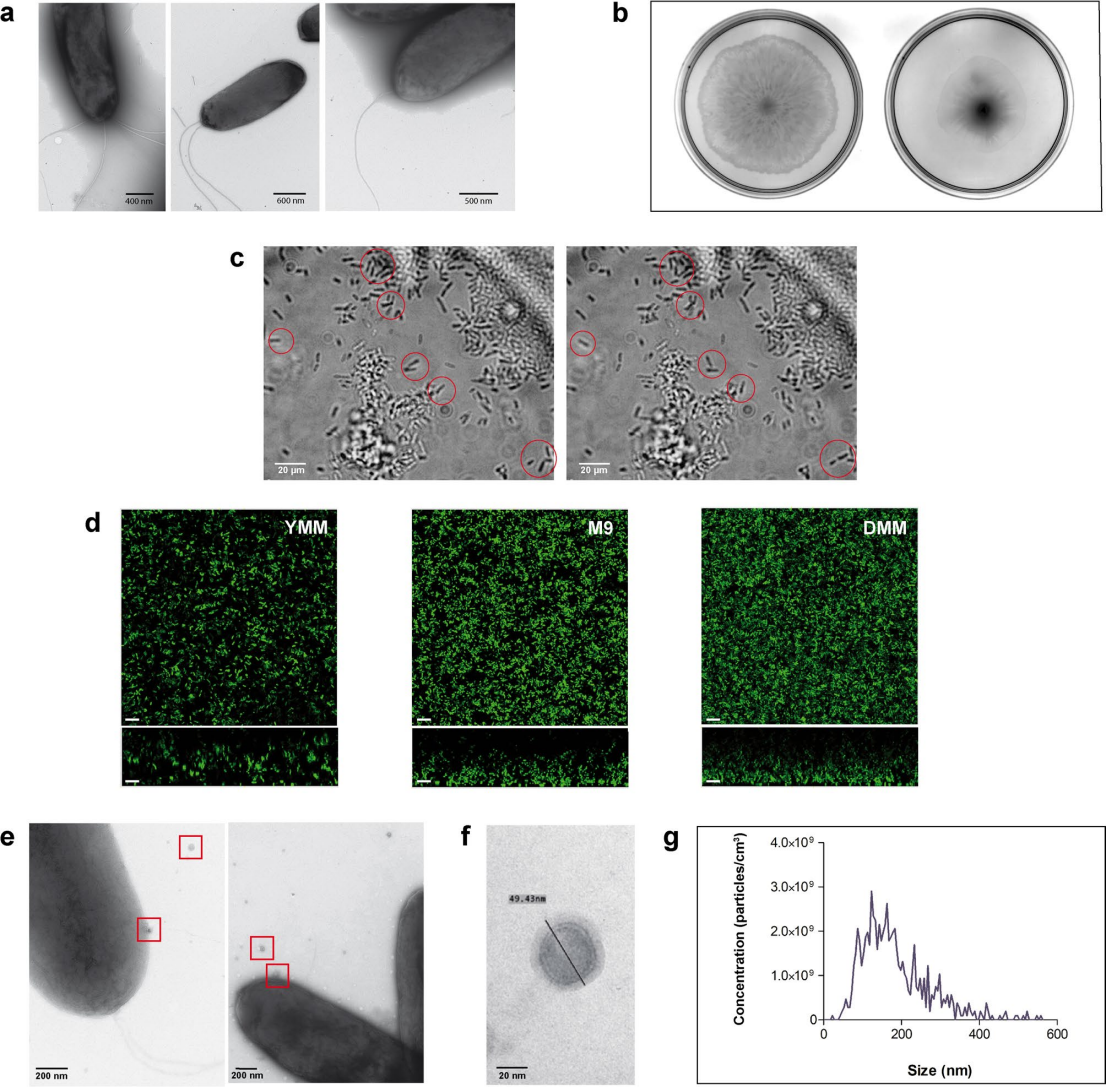
Phenotypic traits of *P. soli* strain VMAP1 related to fitness and biocontrol. (**a**) Transmission electron microscopy (TEM) images of polar flagella. (**b**) Swarming (left; 0.6% agar) and swimming (right; 0.3% agar) patterns on semi-solid media. (**c**) Representative images of twitching (time-lapse optical microscopy, 1000×); red circles indicate cells with tumbling behaviour over a 5 s interval. (**d**) Confocal laser scanning microscopy (CLSM) analysis of biofilm formation under different minimal media conditions (scale bars: 10 µm). (**e**,**f**) TEM images of outer membrane vesicles (OMVs) release and purified OMVs. (**g**) Nanoparticle tracking analysis (NTA) of OMVs size distribution and concentration.

Phenotypic characteristics associated with motility and biofilm formation were also investigated in vitro. Electron microscopy showed that VMAP1 cells possess either a single flagellum or multiple flagella (Fig. 2a) located at one pole, which proved capable of driving both swimming and swarming (Fig. 2b). In addition, individual cells exhibited movements compatible with twitching, which confirms the presence of T4P (Fig. 2c). No biofilm architecture was detectable under confocal laser scanning microscopy (CLSM) after the transformed strain (VMAP1-GFP) was grown on minimal media for 24 or 48 h (Fig. 2d). In other words, this strain was not able to develop mature biofilm under the conditions tested.

The ability of VMAP1 to produce outer membrane vesicles (OMVs) was studied next, since these vesicles are increasingly being recognised as key biocontrol mediators due to their antibacterial and anti-biofilm activity^18,19^. Transmission electron microscopy (TEM) images revealed that spherical particles adhered to some VMAP1 cells or in their proximity (Fig. 2e) were morphologically consistent with OMVs. The OMV fraction purified from the cell-free supernatant (CFS-AP1) was subsequently subjected to TEM observation and nanoparticle tracking analysis (NTA). A representative TEM image is shown in Fig. 2f. According to the NTA, the OMVs concentration in the fraction was 6.7 × 10^9^ particles mL^-1^, with most particles ranging between 111 and 174 nm in size (Fig. 2g).

**Fig. 3:**
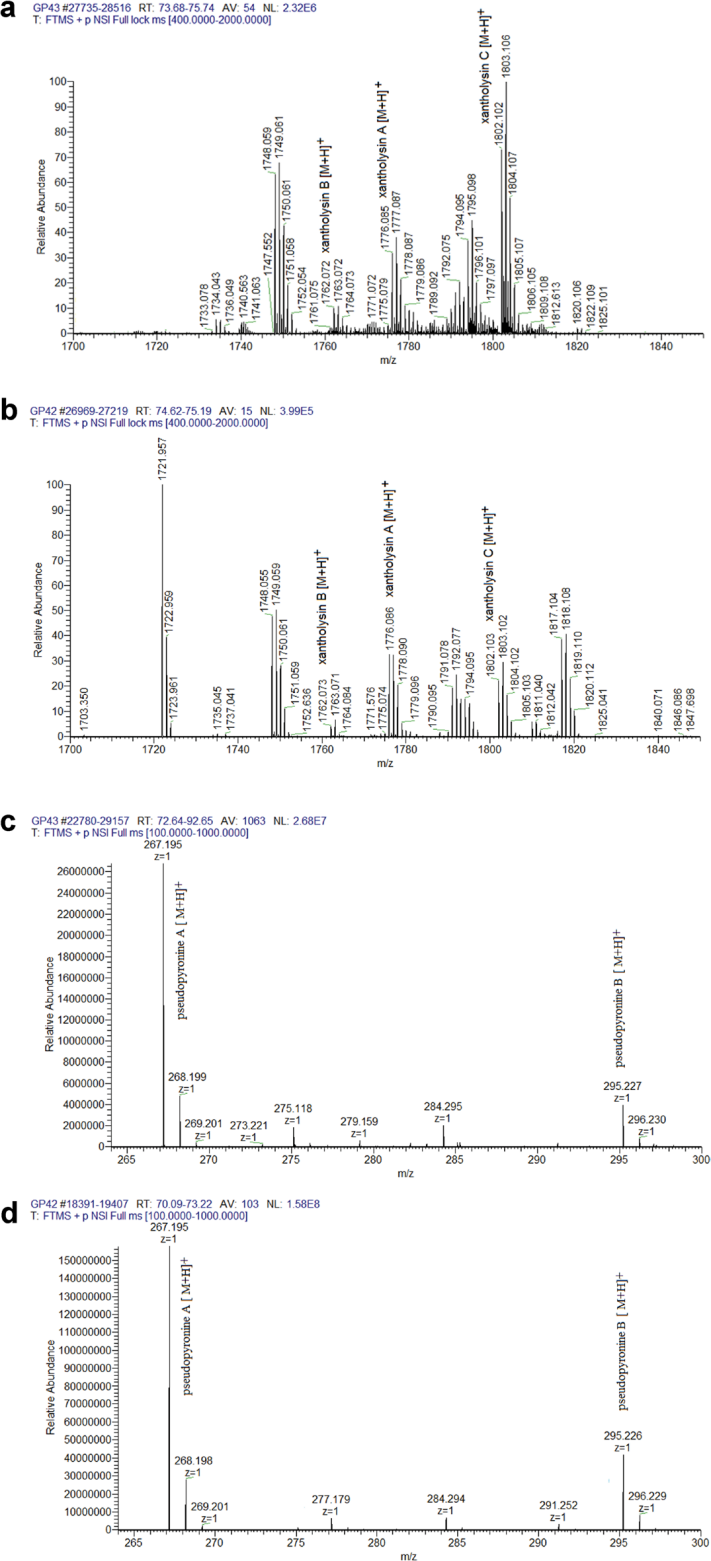
Secondary metabolites produced by *P. soli* strain VMAP1. Electrospray ionisation mass spectra (ESI–MS) showing the detection of xantholysins A-C in (**a**) CFS-AP1 and in (**b**) the purified OMV fraction, and of pseudopyronines A and B in (**c**) CFS-AP1 and in (**d**) the purified OMV fraction.

A series of assays were performed to confirm that VMAP1 produces compounds which have been reported to have biocontrol activity, and for which it harbours the corresponding genes or biosynthetic gene clusters (BGCs). The production of HCN was ascertained in vitro (Fig. S3). Furthermore, mass spectrometry (MS) detected cyclic lipopeptides (CLPs) from the xantholysin family and pseudopyronines in the methanolic extracts from both CFS-AP1 and the purified OMV fraction. Prominent peaks belonging to xantholysins A, B and C (Fig. 3a,b) as well as to pseudopyronines A and B (Fig. 3c,d) were observed through electrospray ionisation mass spectrometry (ESI–MS). The chemical structures of the protonated molecular ions for the three xantholysins were respectively found at m/z 1776.09, m/z 1762.07 and m/z 1802.10 by tandem ESI–MS/MS (Fig. S4–6). Their identification was further supported by diagnostic fragment ions corresponding to each possible xantholysin structure (Table S4). On the other hand, the detection of protonated molecular ions at m/z 267.195 and m/z 295.227 respectively corroborated the synthesis of pseudopyronines A and B, consistent with published data^20^. The low signal intensity of the peaks did not make it possible to acquire fragmentation spectra (MS/MS) for these compounds. Nevertheless, accurate mass measurements obtained using high-resolution Orbitrap MS provided further evidence of their presence.

### Biological activity of metabolites produced by VMAP1

**Fig. 4:**
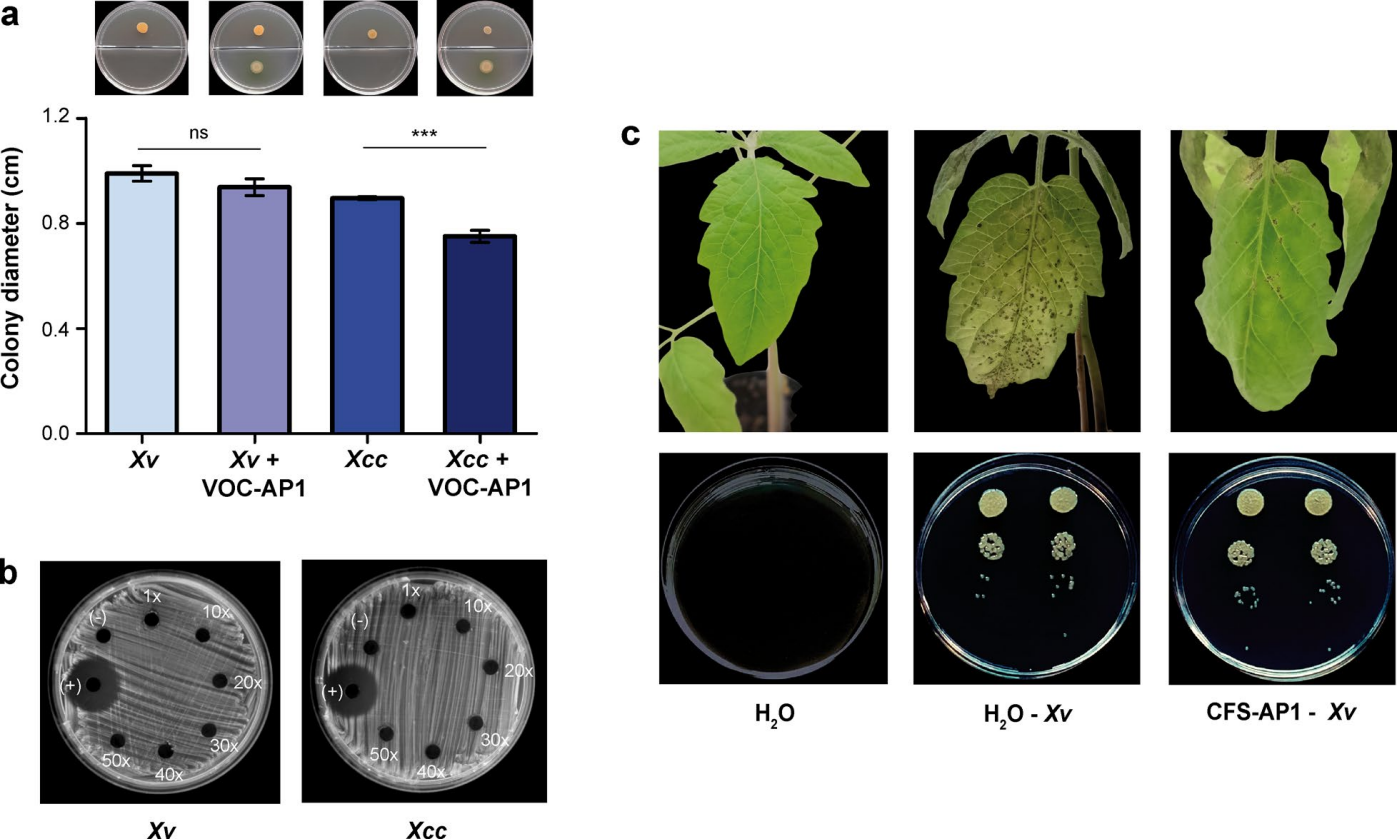
Effects of metabolites produced by *P. soli* strain VMAP1 on *Xanthomonas* spp. viability. (**a**) Growth inhibition mediated by volatile organic compounds (VOCs-AP1). Bars represent mean ± SD. Statistically significant differences (*t*-test, *P* ≤ 0.05) are indicated by “***” and non-significant differences are denoted as “ns”. Representative growth of *Xv* and *Xcc* in the absence or presence of VOCs-AP1 is shown above. (**b**) In vitro antagonistic activity of VMAP1 cell-free supernatant (CFS-AP1) against *Xv* and *Xcc*. Positive (+) and negative (−) controls are indicated. (**c**) Protective effect of CFS-AP1 against *Xv* infection in tomato plants. Top panels show representative tomato leaflets under the following treatments: uninfected control (left), *Xv*-infected plants pre-treated with H_2_O (middle), and *Xv*-infected plants pre-treated with CFS-AP1 (right). Bottom panels show the corresponding *Xv* cells recovered from plant tissue for each treatment.

Volatile organic compounds produced by VMAP1 (VOCs-AP1) showed no inhibitory activity against *Xv*. These compounds included HCN, whose production had been confirmed in vitro (Fig. S3). Their inhibitory potential was thus tested against *X. campestris* pv. *campestris* (*Xcc*), since VOCs synthesised by *P. putida* BW11M1 have been shown to inhibit its growth^21^. Unlike what occurred with *Xv*, VOCs-AP1 significantly inhibited the growth of *Xcc* (Fig. 4a), which suggests that their effectiveness may vary according to the target strain.

The antagonistic activity of diffusible metabolites in CFS-AP1 was also evaluated in vitro against *Xv* and *Xcc.* Increasing concentrations of the supernatant were used, ranging from the original sample up to a 50-fold concentration. *Xcc* was included so as to determine whether any observed inhibitory effects were strain-dependent, as was the case with the VOCs. However, CFS-AP1 had no antagonistic activity against these bacteria, even at the highest concentration tested (Fig. 4b). Similarly, the growth of *Xv* was not inhibited in tomato plants pre-treated with CFS-AP1 in a later experiment (Fig. 4c).

**Fig. 5:**
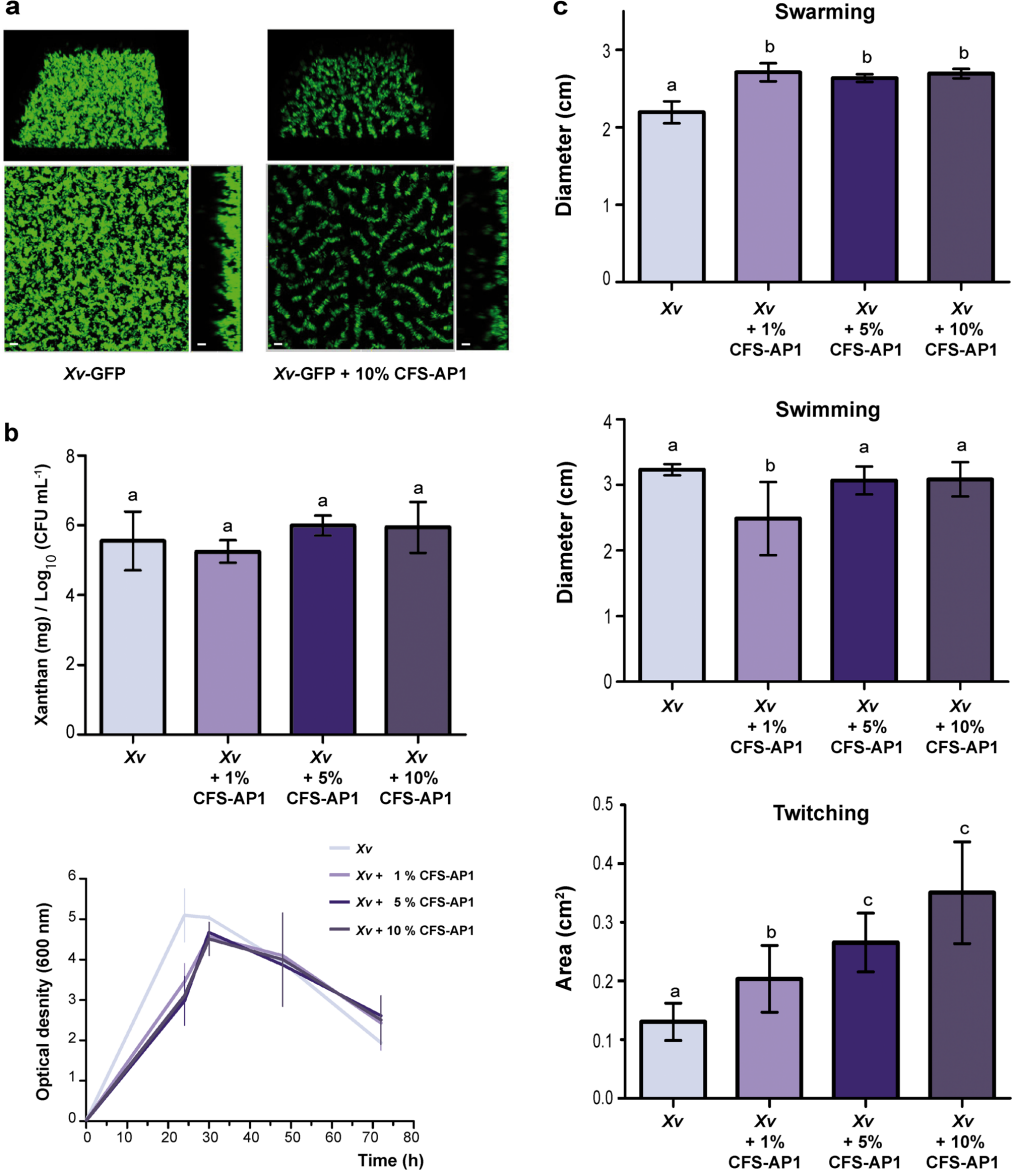
Effects of diffusible VMAP1-derived metabolites on virulence-related traits in *Xv*. (**a**) Biofilm formation by *Xv-*GFP analysed by CLSM (400× ; scale bars: 10 µm) in the absence (left) or presence (right) of 10% CFS-AP1. The *x*–*y* projections are displayed on the left and *z*-axis projections on the right. (**b**) Effects of CFS-AP1 on xanthan production (top) and bacterial growth (bottom). (**c**) Swarming, swimming and twitching by *Xv* under increasing concentrations of CFS-AP1. Bars represent mean ± SD. Different letters indicate statistically significant differences (one-way ANOVA followed by Tukey’s test, *P* ≤ 0.05).

The effects of CFS-AP1 were then studied on the production of virulence factors by *Xv*. As shown in Fig. 5a, exposure to CFS-AP1 interfered with the ability of transformed *Xv* (*Xv*-GFP) to form mature biofilm. In contrast, it did not significantly affect xanthan production (Fig. 5b). Although twitching and swarming increased significantly in the presence of CFS-AP1, the effect was concentration-dependent for twitching (Fig. 5c, bottom) but not for swarming (Fig. 5c, top). Swimming decreased significantly only at the lowest concentration of CFS-AP1 (1%), but was not significantly altered at 5% and 10% with respect to the control (Fig. 5c, middle).

Finally, the effects of diffusible VMAP1-derived metabolites were assessed in planta. The focus was placed on stomatal movements and callose deposition, given that they are two key mechanisms involved in restricting the spread of *Xanthomonas* spp. within the host.

**Fig. 6:**
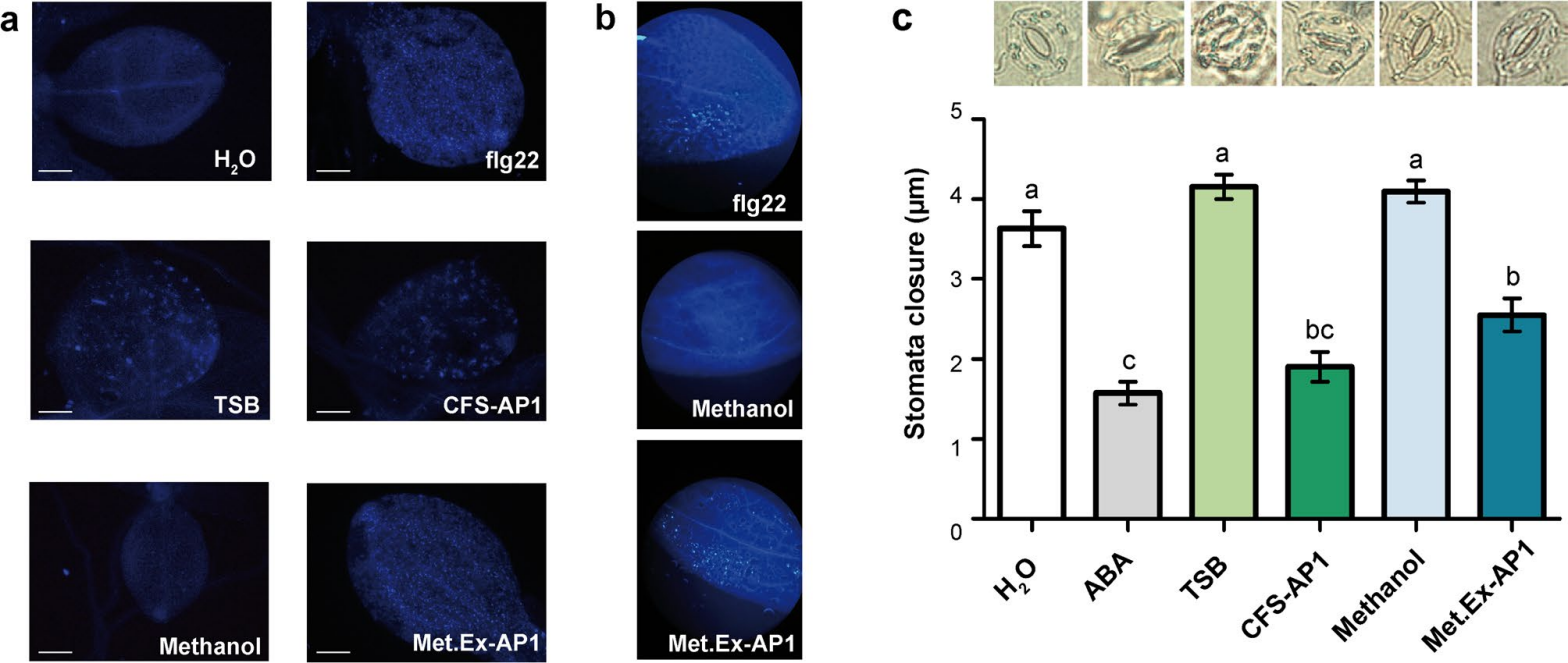
Induction of plant defence responses by diffusible VMAP1-derived metabolites. (**a**) Callose deposition in *A. thaliana* seedlings. Representative optical microscopy images (400× ; scale bars: 20 µm) after treatment with CFS-AP1, Met.Ex-AP1, TSB or methanol. H_2_O and flagellin peptide flg22 were included as negative and positive controls, respectively. (**b**) Callose deposition in tomato seedlings. Representative phenotypes of tomato seedlings treated with Met.Ex-AP1, methanol or flg22. (**c**) Stomatal closure in tomato leaf epidermis. The bar graph shows the stomata closure after treatment with CFS-AP1, TSB, Met.Ex-AP1 or methanol. H₂O and abscisic acid (ABA) were used as negative and positive controls, respectively. Data represent mean ± SD. Different letters indicate statistically significant differences (one-way ANOVA followed by Tukey’s test, *P* ≤ 0.05).

Callose deposition was first studied in *Arabidopsis thaliana* (Fig. 6a), a model system already established in our laboratory, and subsequently validated in tomato (Fig. 6b). Even though CFS-AP1 induced callose deposition in both hosts, a similar effect was registered in the negative control with TSB. To rule out any influence by the culture medium, the experiment was carried out with methanol from the organic fraction of the supernatant (Met.Ex-AP1), in which we had detected CLPs from the xantholysin family and pseudopyronines. This also triggered callose deposition, whereas methanol alone (used as a solvent control) had no effect.

When stomatal closure was examined in tomato, both CFS-AP1 and Met.Ex-AP1 were found to significantly induce it with respect to the controls with TSB and methanol. The stomatal response to CFS-AP1 did not differ significantly from that of the positive control with abscisic acid (ABA). Although Met. Ex-AP1 induced a slightly lower degree of stomatal closure than ABA did, its effect was statistically similar to that of CFS-AP1 (Fig. 6c).

## Discussion

Several *Pseudomonas* spp. are known to synthesise a vast array of metabolites with plant growth-promoting properties, antimicrobial activity, and/or the ability to induce systemic resistance in plants. However, *P. soli* is among those strains whose biocontrol potential has not been sufficiently studied at the genomic and functional levels. Here, we integrated in silico, in vitro and in planta analyses to characterize the physiological traits of *P. soli* strain VMAP1. The ultimate aim was to provide a comprehensive framework to understand its mechanisms of action and its applicability as a biological control agent.

We first confirmed the phylogenetic status of VMAP1 as a member of *P. soli*. This is particularly relevant given that numerous *Pseudomonas* strains have been misclassified due to the use of inadequate taxonomic methods. For instance, strains FBJ P5B2 and T13GI4 were assigned to *P. soli* based solely on 16S rRNA sequence identity^11,12^, yet the commonly applied threshold for identity with this analysis (97–98.7%) only makes it possible to delineate broad taxonomic groups within *Pseudomonas.* Whole-genome comparisons have in fact proven that strains sharing over 98–99% identity may still represent distinct species within the genus^22^. On the other hand, those strains which have been correctly classified as *P. soli*, such as RUB1 and CCOS 191, have not been subjected to in vitro or in planta studies that may demonstrate their antagonistic, biocontrol or plant growth-promoting activity^23^. VMAP1 was shown in our earlier research to suppress *Xv* infection in planta^13^*,* and as such remains to date the only *P. soli* strain whose biocontrol capacity has been experimentally validated.

When its genome was functionally annotated in the present study, the emerging enriched COG categories were related to amino acid metabolism, transcription, signal transduction and cell envelope biogenesis. These genomic features have been previously identified as hallmarks of ecological plasticity and metabolic versatility in *Pseudomonas*^24,25^, and they suggest that VMAP1 possesses the genomic toolkit required for nutrient acquisition, environmental sensing and structural adaptability typically associated with biocontrol agents.

The genome also features clusters responsible for alginate and Pap biosynthesis, but lacks the genes necessary for producing Pel, Psl, PNAG, levan, cellulose, Pea, and Peb. In beneficial plant-associated *Pseudomonas*, alginate provides viscosity and mechanical strength, while Pap contributes to cell cohesion and resistance to surfactants. The other EPSs are involved in modulating biofilm dynamics and providing energy reserves, mechanical strength and structural redundancy^26,27^. The limited repertoire of EPS genes in VMAP1 may partly explain why this strain failed to form a mature biofilm in subsequent in vitro experiment, a trait associated with persistence and beneficial interactions with plants in other biocontrol *Pseudomonas.* Nevertheless, the genomic data suggest this strain may not rely on canonical EPS matrices for colonisation. It may use alternative adhesion mechanisms or require specific signals from the rhizosphere or phyllosphere to establish stable sessile communities. This would not be unlikely for a member of a genus known for its diversity and adaptability, but will require corroboration through competitive colonisation studies.

Moreover, VMAP1 was later observed to produce OMVs (OMVs-AP1) in vitro. In other bacteria, these vesicles have been implicated in the delivery of bioactive molecules to the extracellular space, and they are increasingly recognised as key mediators of microbial competition, communication, host interactions and biocontrol^18,19^. In the absence of biofilm-forming ability as a canonical colonisation determinant, the production of OMVs by VMAP1 may represent a specialised mechanism for the deployment of antimicrobial strategies and enhanced competitive fitness in plant environments.

Other elements in the genome seemingly associated with colonisation and interbacterial competition include CDSs homologous to the *gacS*/*gacA* two-component system (a master regulator which additionally controls secondary metabolite production)^9^, as well as T6SS-related components. However, the low sequence identity observed for some of these components (e.g. *tssL* and *clpV*) combined with the absence of genes encoding PAAR-domain proteins point to significant evolutionary divergence from better-characterized systems^28^. Such variations call into question the functional status of the T6SS in VMAP1, potentially reflecting either a vestigial state or an unconventional architecture adapted to its ecological niche. Functional assays of effector secretion or antibacterial activity would offer a more definitive answer to whether T6SS effectively contributes to niche establishment or microbial interference in plant-associated VMAP1.

Lastly, certain key genomic features were identified which may contribute to the ability of VMAP1 to suppress phytopathogens. These include genes and gene clusters involved in the biosynthesis of pseudopyronines, CLPs from the xantholysin family, HCN, lytic enzymes, PQQ and a putative R-type pyocin.

The in silico analysis of the genome was complemented with a series of phenotypic assays in vitro. They demonstrated that VMAP1 tolerates a broad range of environmental conditions (temperature, pH, salinity), is resistant to ampicillin and chloramphenicol, and displays enzymatic activity. Its motility was observed to be mediated by polar flagella and T4P, which may facilitate access to and colonisation of spatially distant niches.

In phenotypic assays, VMAP1 was able to produce many of the bioactive metabolites for whose synthesis genes had been located in the genome. Both CFS-AP1 and OMV-AP1 contained pseudopyronines A and B as well as xantholysins A, B and C. This suggests the existence of multiple secretion mechanisms and supports the VMAP1 potential as a biocontrol agent. Pseudopyronines are α-pyrones that disrupt bacterial membrane integrity and inhibit key enzymes involved in fatty acid biosynthesis. They have shown broad-spectrum antimicrobial and anti-biofilm activity^20,29,30^. CLPs from the xantholysin family similarly affect cellular membrane integrity and strongly antagonise fungi and gram-positive bacteria. Xantholysin A in particular is also active against certain gram-negative bacteria^21,31,32^. Thus, these diffusible metabolites can contribute to the biocontrol activity previously reported for CFS-AP1^13^.

Though not functionally assessed here, two diffusible metabolites encoded in the VMAP1 genome may further contribute in a relevant manner to the strain’s biocontrol ability, and thus merit additional inquiry at the functional level. These are PQQ and R-type pyocins. The former is a redox cofactor involved in phosphate solubilisation, the regulation of antimicrobial compound synthesis and the induction of plant systemic resistance^33^. For their part, R-type pyocins are contractile bacteriocins that kill bacteria by membrane disruption and nucleic acid degradation^34,35^. In VMAP1, they may be indicative of a specialized capacity for targeted bacterial competition. Although some studies have reported their bactericidal activity^36–38^, they remain underexplored in plant-beneficial pseudomonads.

VMAP1 also produced HCN*,* a well-characterised compound known for its activity against phytopathogens and its capacity to promote plant growth^6,7,9^. Although HCN and other VOCs-AP1 failed to inhibit the growth of *Xv *in vitro under the chosen conditions, they significantly reduced the growth of *Xcc.* This pathogen-specific response may be linked to compound concentration and/or pathogen sensitivity. Strain-dependent VOC efficacy was similarly reported by Almeida and colleagues^39^.

In a previous study, we found that the foliar application of CFS-AP1 in tomato plants reduced bacterial spot severity by 75%, whereas irrigation with living VMAP1 cells resulted in a 44% reduction^13^. However, in the present study, the diffusible metabolites in CFS-AP1 failed to inhibit *Xv* growth in vitro, even when concentrated 50-fold, suggesting that insufficient metabolite levels are unlikely to explain the lack of growth inhibition. Accordingly, there was no significant reduction in the viable cell counts of *Xv* populations recovered from tomato leaves which had been pre-treated in planta with CFS-AP1, as mentioned before. Both findings suggest that rather than operate through direct bactericidal activity, VMAP1 may predominantly affect pathogen virulence and modulate the host immune response. For further elucidation on this point, specific assays were conducted to assess the effects of diffusible VMAP1-derived metabolites on *Xv* virulence factors and plant defence responses.

*Xanthomonas* spp. rely on a programme that coordinates motility, surface attachment and biofilm formation to transition from host entry to stable colonisation^40^. Even partial disruption of such a programme, particularly during the early stages of infection, can irreversibly compromise *Xv* pathogenesis even when cell viability remains unaffected^41^. Here, we observed that CFS-AP1 significantly increased *Xv* swarming and twitching while simultaneously impairing biofilm formation. Collectively, these results may reflect a disruption of the transition from a planktonic to a sessile lifestyle in *Xv*. Maintenance of the pathogen in a hyper-motile state would be expected to hinder stable surface colonisation, ultimately compromising biofilm establishment and maturation.

Interestingly, even though xanthan is a key structural component of the *Xanthomonas* biofilm matrix^42^, its production by *Xv* was not modified in this study when grown in the presence of CFS-AP1. The observed disruption in biofilm formation may thus have occurred through other means, such as interference with extracellular enzymes, adhesion proteins or cell surface interactions. This could be possible thanks to the surfactant or proteolytic activity of some VMAP1-derived metabolites^43,44^, as is the case of the xantholysins detected in both CFS-AP1 and the OMV-AP1 fraction. Anti-biofilm activity has in fact been reported for other CLPs (e.g., viscosinamide, massetolide and putisolvin) in similar systems^45^. Nonetheless, experimental data on xantholysins are scarce and a fuller understanding of their role in this pathosystem may be gleaned through targeted mutagenesis assays. Pseudopyronines A and B, which were detected in CFS-AP1 and OMV-AP1 in this study, may likewise be involved in the ability of VMAP1 to upset *Xv* biofilm formation, since they have been shown to degrade mature biofilms in other species^29^. Moreover, VMAP1 has been reported to produce protease^13^, an enzyme that could further weaken biofilm cohesion and structural integrity by degrading matrix-associated proteins.

Lastly, callose deposition was induced in planta (both in *A. thaliana* and tomato leaves) by CFS-AP1 and Met.Ex-AP1. The CFS-AP1 also induced stomatal closure in a similar manner to ABA, a well-characterised phytohormone responsible for this phenotype. The effect of Met.Ex-AP1 on this parameter was slightly less pronounced but not significantly different from that of CFS-AP1. Considering that these plant immune responses are known to limit bacterial entry and proliferation in planta^46,47^, the results suggest that the diffusible compounds produced by VMAP1 may also be capable of activating plant immunity. They could additionally contribute to explaining the efficacy of foliar application of CFS-AP1 in reducing bacterial spot severity in tomato plants, as previously reported^13^.

Together, the data presented here support the notion that VMAP1 interrupts *Xv* infection instead of directly eliminating the pathogen, in agreement with models of host-mediated disease suppression proposed for other beneficial microorganisms^48,49^. The distinction is not a trivial one, as the efficacy of biocontrol by *Pseudomonas* spp. has traditionally been linked to the production of antibiotics such as 2,4-diacetylphloroglucinol (DAPG), phenazines or bactericidal CLPs^50,51^. Admittedly, several well-characterised *Pseudomonas* strains synthesise metabolites that combine antimicrobial activity with the ability to stimulate plant immune responses, although the relative contribution of each mechanism varies depending on the host–pathogen system. For instance, DAPG produced by *P. fluorescens* CHA0 induces callose deposition^51^, and phenazines and CLPs produced by *Pseudomonas* sp. CMR12a are key determinants of induced systemic resistance (ISR)^52^. In contrast, our results show that effective disease suppression can occur without detectable pathogen growth inhibition. Anti-virulence strategies and immune modulation thus emerge as biologically significant and complementary pathways beyond simple antibiosis, though the molecular mechanisms underlying these abilities in VMAP1 should be clarified by transcriptomic, genetic, and signalling-based analyses.

In this context, the absence of biosynthetic pathways for DAPG and phenazine in the VMAP1 genome, alongside its production of CLPs from the xantholysin family and pseudopyronines, point to the deployment of a distinct functional strategy. These metabolites have been primarily investigated for their antimicrobial or antibiofilm activity, but their involvement in plant defence activation warrants further investigation. What is more, they were identified within the OMVs-AP1, which hints at the possibility of these vesicles being able to enhance the spatial stability, integrity or targeted delivery of the bioactive molecules. Future research should assess purified OMV-AP1 in terms of its functional relevance in the plant-pathogen interaction.

From an applied perspective, the predominance of anti-virulence and plant defence induction mechanisms in VMAP1-mediated biocontrol may have important implications. Unlike traditional antimicrobials used in agriculture, these strategies do not exert bactericidal pressure which favours directional selection in pathogen populations, and might thus reduce the emergence of resistance^53^. Furthermore, their effectiveness may be longer-lasting in the field, where ecological complexity and microbial competition often impair the performance of purely bactericidal agents. Still, experimental evolution studies are needed to determine whether these modes of action effectively translate into a lower incidence of resistance under real-world conditions^54^.

Overall, the present study found that *P. soli* strain VMAP1 coherently integrates genomic potential, phenotypic versatility and functional activity against *Xv*. Its repertoire of metabolites which induce plant immunity and interfere with *Xv* virulence, coupled with its ability to produce OMVs which may be involved in the release and targeted delivery of these compounds, contribute to its profile as a promising candidate for the sustainable management of bacterial spot in tomato. While these functional and mechanistic observations were made under controlled conditions, the biocontrol activity of VMAP1 has also been previously demonstrated in greenhouse trials^13^. Therefore, in addition to all the other avenues open for ulterior research mentioned thus far, future studies should prioritise field-scale validation and performance assessments in consortia with other PGPR strains. The comprehensive picture thus obtained will likely make it possible to use VMAP1 as the basis for robust, resilient and environmentally compatible biocontrol formulations.

## Methods

### Genome sequencing, de novo assembly and annotation

The DNA of VMAP1 was obtained with the Wizard® Genomic DNA Purification Kit (Promega, WI, USA) following the manufacturer’s instructions. Its quantity and quality were determined with a NanoDrop One Microvolume UV/Vis spectrophotometer (Thermo Fisher Scientific) and 0.8% agarose gel electrophoresis.

The genome was sequenced at Macrogen Inc. (NGS Service, Seoul, Korea) on the Illumina HiSeq 2500 platform (2 × 100 bp paired end reads). The quality of the obtained reads was examined on FastQC v.0.11.8 (Babraham Bioinformatics, Cambridge, UK). The raw reads were quality filtered and trimmed on Trimmomatic v.0.38 (parameters: ILLUMINACLIP: TruSeq3-PE.fa:2:30:10, LEADING:3 TRAILING:3 SLIDINGWINDOW:4:15 MINLEN:36)^55^.

De novo assembly was performed on A5-miseq^56^, ABySS^57^, DISCOVAR^58^, MaSuRCA^59^ and SPAdes^60^. The assemblies were then combined into a single superior assembly on Metassembler^61^. This software merges assemblies in an iterative manner, i.e., the best local sequences in the first pair to be merged are the basis for improving the next iteration or pair of assemblies. For this reason, the six de novo assemblies were manually ranked beforehand according to their N50 size. Scaffolding was carried out on MeDuSa^62^ with the five closest genomes from NCBI as references (GCF_000498975.2, GCF_003205305.1, GCF_003205475.1, GCF_009914935.1, GCF_900110655.1).

The curated genome sequence was annotated using the NCBI Prokaryotic Genome Annotation Pipeline (PGAP)^63^ and deposited in GenBank under the accession number GCA_025643155.1. The assembled genome and the raw sequencing reads are available under BioProject PRJNA758981.

### Identification at the species level using genomic data and gene mining

To confirm the taxonomic classification of VMAP1, average nucleotide identity (ANI) was established on FastANI^64^ between the sequenced output of the pipeline and 16 other *P. soli* genomes available at GenBank. The concatenated sequences *rpoD*^346–1196^-*pepN*^1711–2571^-*gltX*^86–909^ were used for multilocus sequence analysis (MLSA) on Molecular Evolutionary Genetics Analysis v11 (MEGA11)^65^. These loci were selected due to their superior phylogenetic robustness, high sensitivity and complementarity to ANI, as reported by Garavaglia and colleagues^66^. Protein-coding sequences were assigned to Clusters of Orthologous Groups (COG) categories on GenoVi^67^.

Gene clusters involved in secondary metabolite production were predicted with the antiSMASH annotation tool (version 7.1.0) using default parameters. BGCs were detected with PRISM, NaPDoS2 and PKS/NRPSas backups. Their identity was confirmed by inputting the predicted non-ribosomal peptide (NRP) sequences in the VMAP1 genome into the NORINE NRP database. Further corroboration was obtained on BLASTP (stand-alone v. 2.14.0 +). A minimum 80% sequence identity and coverage was necessary for any given gene to be deemed present. Sequences falling below this threshold were thus considered absent. Operon presence/absence was ascertained depending on the presence/absence of most of the constituent genes. Most of the CDSs analysed were compared with gene or amino acid sequences identified in *Pseudomonas* spp. which are phylogenetically close to VMAP1, such as *P. putida*, *P. mosselli* and *P. entomophila.* Nevertheless, some CDSs were aligned with genes described only in distantly related species, such as *P. aeruginosa* and *P. fluorescens*. Additional genes of interest were identified through stand-alone BLASTP searches (v. 2.14.0 +).

### Bacterial strains and general culture conditions

*P. soli* strain VMAP1 was originally isolated from the rhizosphere of healthy tomato plants, as described by Felipe et al.^13^. It is listed in Table 2 alongside all the other strains used in this study. Unless otherwise specified, these strains were routinely grown on tryptone soy agar (TSA) or in tryptone soy broth (TSB) and incubated at 28 °C and 200 rpm for liquid cultures. A complete list of the culture media used in this study can be found in Table S5.

**Table 2:** Bacterial strains and plasmids used in this study.

Strain/plasmid	Characteristics	Reference
VMAP1	*Pseudomonas soli* isolated from rhizosphere of healthy tomato plants	^13^
VMAP1-GFP	*P. soli* strain VMAP1 carrying pBBR2-GFP	This study
*Xv*	*Xanthomonas vesicatoria* wild type strain BNM 208	^75^
*Xv*-GFP	*X. vesicatoria* wild type strain BNM 208 carrying pBBR2-GFP	^75^
*Xcc*	*X. campestris* pv. *campestris* wild type strain 8004	^76^
*Escherichia coli* S-17/pBBR2-GFP	*E. coli* 294 thi RP4-2-Tc::Mu-Km::Tn7 integrated into chromosome carrying the pBBR2-GFP vector	^42^
pBBR2-GFP	Broad-host-range plasmid pBBR1MCS-2 expressing kanamycin resistance cassette and the green fluorescent protein gene (*gfp*) constitutively	^77^

### Growth dynamics and antibiotic resistance

The growth of VMAP1 was assessed at different temperatures, pH values and NaCl concentrations. To evaluate the effects of temperature (20, 28, 37, 40 and 45 °C), VMAP1 was grown at pH 7.2 with 0.5% (w/v) NaCl. The effects of pH (values between 2 and 12) were analysed at 28 °C, in TSB supplemented with 0.5% (w/v) NaCl. Finally, the effects of NaCl concentration (0.5–16% w/v) were subsequently studied at 28 °C and pH 7.2. In all cases, the cultures were incubated for 96 h in TSB with 200 rpm agitation. Samples were collected at 24-h intervals to quantify optical density at 600 nm (OD_600_) and the number of colony-forming units per millilitre (CFU mL^-1^).

To assay antibiotic susceptibility in vitro, VMAP1 was grown on TSA and Müeller-Hinton agar (MHA, Table S5) supplemented with ampicillin (8, 32 and 200 µg mL^-1^), tetracycline (5 µg mL^–1^), kanamycin (4, 16 and 50 µg mL^–1^), gentamicin (4, 16 and 30 µg mL^–1^), streptomycin (4, 16 and 30 µg mL^–1^), polymyxin B (2, 8 and 30 µg mL^–1^) or chloramphenicol (8, 32 and 35 µg mL^–1^). One hundred µL were subsequently taken from suspensions made from 24-h cultures (OD_600_ = 1) and spread on agar plates. Sterile filter paper discs (5 mm in diameter) were soaked in 10 µL of each antibiotic and placed on top of each plate. The plates were incubated for 24 h at 28 °C. The inhibition zones were measured three times, and the means and standard deviations were calculated.

### Preparation of cell-free supernatant

A cell-free supernatant (CFS-AP1) was obtained from a VMAP1 culture in TSB, which was incubated for 24 h at 28 °C and 200 rpm. The cells were removed by centrifugation (18,000 × *g* for 1 h at 4 °C). The supernatant was put through PVDF syringe filters (0.22 μm pore size) and 10 μL were plated on TSA and incubated at 28 °C for 96 h to confirm sterility.

### Presence and functionality of flagella and T4P

Flagella were observed by TEM. Negative staining was performed as previously described^68^ with some modifications. VMAP1 was grown statically in TSB at 28 °C for 8 h. Formvar-coated nickel grids were then floated for 10 min on 15 µL of bacterial suspension. The grids with absorbed bacteria were incubated in a 2% phosphotungstic acid solution for 1 min and rinsed with distilled water. Excess liquid was drained off with the edge of a filter paper. The preparations were air-dried for 10 min, examined under a Hitachi HT7800 electron microscope (Hitachi, Tokyo, Japan) operated at 80 kV, and photographed with a NanoSprint15 AMT camera (Advanced Microscopy Techniques Corp., Woburn, MA, USA).

To examine flagellar functionality, swarming and swimming assays were carried out as described previously^69^ with some modifications. Two µL of a bacterial suspension (OD_600_ = 0.01) were placed in the centre of 90 mm Petri dishes containing either King B (Table S5) with 0.6% (w/v) agar for swarming, or LB (Table S5) with 0.3% (w/v) agar for swimming. The plates were incubated at 28 °C for 18 and 20 h to observe swarming, or for 24 and 30 h to observe swimming.

An earlier protocol^70^ was adopted to analyse T4P functionality by observing twitching under an optical microscope. As a preliminary step, King B medium was prepared with 1% (w/v) agar and supplemented with 2 mM CaCl_2_. Then, the upper surface of a glass slide was entirely coated with double-sided adhesive tape (clear 3 M VHB, 19 mm wide, 1 mm thick). A piece in the shape of a rectangle was removed from the center with a scalpel, and the space left was filled with melted King B (as prepared in the preliminary step). Another glass slide was placed on top, in order to allow the medium to solidify into a flat and smooth surface. Once this was achieved, the top slide was carefully removed and the first slide was seeded with 2 µL of a VMAP1 suspension, which had been prepared in sterile water from a TSA plate incubated overnight. The slide was left to dry at 28 °C for 30 min and a cover glass was placed on top for microscopic observation under a Nikon Eclipse E600. The movement of individual cells was monitored, and images were captured every 5 s using a Lanoptik MC500W-G1 digital camera.

### OMV purification, detection, and quantification

The OMVs-AP1 were obtained from VMAP1 cultures. Briefly, the strain was incubated at 28 °C and 200 rpm in a 1 L flask containing 150 mL of TSB for 24 h. The cells were pelleted at 9,000 × *g* for 20 min at 4 °C. The supernatant was collected, centrifuged again at 9,000 × *g* for 20 min, and filtered through 0.45 μm and 0.22 μm PES membranes (Membrane Solutions) to remove residual cells. The OMVs-AP1 were recovered from the supernatant by two ultracentrifugation steps, the first at 30,000 × *g* and the second at 180,000 × g, for 2 h each time. The resulting pellet was resuspended in 150 μL of 1% PBS (pH 7.0) and stored at -20 °C. Its sterility was confirmed by incubating a 3 μL drop on a TSA plate for 48 h at 28 °C.

The OMVs-AP1 production was observed by TEM. An aliquot of the purified OMVs-AP1 was stained with a 2% uranyl acetate solution for 3 min and observed under a Zeiss EM 109 T transmission electron microscope. Photographs were taken with a Gatan ES1000W digital camera. Finally, the OMVs-AP1 were quantified with a ZetaView PMX-230 Twin Laser nanoparticle tracker analyser (Particle Metrix).

### Biofilm formation by VMAP1

The ability of VMAP1 to form biofilm was assessed in vitro in a static system. The strain was transformed by conjugation using *E. coli* S17-1/pBBR2-GFP (Table 2), as previously described^71^. The transformed product (VMAP1-GFP) was grown at 28 °C on media supplemented with 30 µg mL^-1^ kanamycin. The cultures were diluted to a final OD_600_ of 0.0005 in YMM, M9 and DMM (Table S5). A total of 500 µL of each bacterial suspension was transferred to chambered cover-glass slides featuring a 1 mm layer of borosilicate glass (LabTek, Nunc, Penfield, NY, U.S.A.). The slides were incubated under static conditions for 72 h at 28 °C in a humid chamber. Biofilm formation was monitored at 24, 48 and 72 h by CLSM under an inverted Nikon Eclipse TE 2000-E2 CLSM (Melville, NY, US). Three-dimensional images were generated using Fiji (ImageJ, version 1.54x; NIH, USA)^72^ with the 3D Viewer.

### Secondary metabolite detection and identification

To ascertain whether VMAP1 produces HCN, it was cultured on King B containing 1% (w/v) agar and supplemented with 4.4 g L^–1^ glycine. The plates were sealed with parafilm and incubated at 28 °C for 72 h. The formation of HCN was indicated by the alkaline sodium picrate Whatman filter paper turning from yellow to reddish brown^6^.

On the other hand, CLPs and pseudopyronines were isolated by the acid precipitation method^73^. CFS-AP1 and the purified OMV fraction were acidified to a final pH of 2 by adding 3 N HCl. They were allowed to precipitate at 4 °C overnight, and the respective precipitates were centrifuged at 5000 × *g* for 30 min at 4 °C. The resulting pellets were resuspended in methanol to extract the CLPs and pseudopyronines (Met.Ex-AP1). Methanol from the organic fractions was evaporated using a vacuum concentrator (Eppendorf Concentrator 5301, Eppendorf AG, Hamburg, Germany), and the samples were stored at –20 °C until needed for the ESI–MS analysis.

The samples were subsequently resuspended in methanol, desalted with ZipTip C18 columns (Millipore), and analysed in a nanoLC-MS/MS in a Thermo Scientific Q Exactive Mass Spectrometer coupled to a nanoHPLC EASY-nLC 1000 (Thermo Scientific). For this, they were eluted for 85 min using a reverse Easy-Spray Column PepMap RSLC-P/N ES801 (C18, 2 μm, 100 A, 50 μm × 150 mm). The flow rate was 3 μL min^-1^, the solvent was acetonitrile:H_2_O and the injection volume was 4 μL. The MS device has a high collision dissociation cell (HCD) for fragmentation and an Orbitrap analyser (Thermo Scientific, Q-Exactive). Electrospray ionization was conducted at 3.0 kV (Thermo Scientific, EASY-SPRAY). Data were acquired on XCalibur 3.0.63 (Thermo Scientific). The scanned mass ranges were 400–2000 m/z and 100–1000 m/z. The 15 most intense ions in each cycle were sequentially isolated and fragmented by HCD, and MS/MA spectra were measured in the Orbitrap analyser. Negative controls were prepared with PBS buffer (pH 7.0) and TSB.

### Antagonistic and/or biocontrol activity in vitro

The activity of VOCs produced by VMAP1, including HCN, was analysed on Petri dishes divided in halves. One half was filled with PYM (Table S5), and the other with King B containing 1% (w/v) agar and supplemented with 4.4 g L^–1^ glycine to induce VOC production. Then, 2 μL of a suspension (OD_600_ = 0.5) of pathogenic bacteria (*Xv* or *Xcc*) were seeded on the PYM side, while 2 µl of a VMAP1 suspension (OD_600_ = 0.2) were seeded on the King B side. The plates were sealed with parafilm to prevent VOC leakage and incubated at 28 °C for 7 days. The cultures were observed every 24 h.

To test the antagonistic activity of CFS-AP1, aliquots of the supernatant were concentrated 10x, 20x, 30x, 40x and 50x using a vacuum concentrator (Eppendorf Concentrator 5301, Eppendorf AG, Hamburg, Germany). In parallel, plates containing 15 mL of TSA were swabbed with 200 μL of an *Xv* suspension (OD_600_ = 1). After they had been left to dry for 2 h at room temperature, 5 mm diameter wells were made in the center and inoculated with 30 μL of the different CFS-AP1 concentrations. The cultures were incubated at 28 °C for 48 h. Antagonistic activity was indicated by a clear zone of pathogen growth inhibition around the wells. Each treatment was replicated twice, and the assay was repeated three times.

CFS-AP1 was also evaluated in terms of its ability to interfere with biofilm formation by *Xv*. For this, a static system consisting of 8-well borosilicate microplates was implemented. *Xv*-GFP (Table 2) was cultured in TSB for 3 h at 28 °C and 200 rpm. Then, bacterial suspensions were diluted in YMM (Table S5) at initial OD_600_ values of 0.005 and 0.001. Aliquots (450 µL) were transferred to chambered cover-glass slides featuring a 1 mm layer of borosilicate glass (LabTek, Nunc, Penfield, NY, U.S.A.), and 50 µL of 1x CFS-AP1 were added to each well. The control was prepared with 450 µL of the same bacterial suspensions and an additional 50 µL of YMM. The cultures were grown in a humid chamber for 72 h at 28 °C. Biofilm formation was monitored by CLSM using the same equipment described above. Three-dimensional images were generated using Fiji (ImageJ) ^72^ with the 3D Viewer.

The effect of CFS-AP1 on xanthan production by *Xv* was assessed as previously described^40^ with minor modifications. Briefly, *Xv* cultures were prepared in 500 mL flasks containing 100 mL of PYM with 2% glucose and supplemented with 1%, 5% or 10% (v/v) CFS-AP1. The cultures were incubated at 28 °C and 200 rpm until the stationary growth phase, i.e. for 72 h. For the control, *Xv* was grown in the same medium and under the same conditions, but in the presence of 10% (v/v) TSB instead of CFS-AP1. At the end of the incubation, the OD_600_ of each culture was measured to determine the relationship between xanthan production and bacterial growth. The bacterial cells were subsequently removed by centrifugation at 25,000 × g for 1 h at 4 °C. Xanthan was precipitated with two volumes of ethanol containing 1% (w/v) KCl and recovered by filtration through a stainless-steel sieve. The wet xanthan fibres were air-dried at room temperature and weighed to determine yield.

Lastly, CFS-AP1 at 1%, 5% or 10% (v/v) was evaluated for its effect on *Xv* motility. Swarming and swimming were studied qualitatively, by examining the size of the halo formed by *Xv* cells grown on 0.5% PYM agar (swarming) or 0.25% NYGB (Table S5) agar (swimming) supplemented with the different concentrations of CFS-AP1. The assay was performed in triplicate as previously described^69^ with some modifications. An earlier protocol was also followed to assay twitching^74^, with minor modifications. *Xv* was grown in TSB supplemented with the different concentrations of CFS-AP1. Single colonies of each culture were then inoculated by stabbing into the bottom of sterile 90 mm Petri plates coated with King B containing 1% (w/v) agar and supplemented with 2 mM CaCl_2_. The plates were incubated at 28 °C for 72 h. After removing the agar, the plates were stained for 30 min with 1% crystal violet and carefully washed with sterile H_2_O. The twitching halos were measured using ImageJ software^72^.

### Antagonistic and/or biocontrol activity in planta

The effect of CFS-AP1 on *Xv* viability was studied in vivo*.* To this end, tomato seeds (hybrid Ichiban, purchased from Bayer CropScience), were grown in individual 1 L pots filled with a commercial substrate (Grow Mix MultiPro, Terrafertil). Only those displaying two to three expanded leaves were then used for the experiment. These leaves were sprayed with CFS-AP1 (1x) until they were dripping, and three days later they were infected with the pathogen. More precisely, they were dipped in a suspension of *Xv* (10^7^ CFU mL^−1^) in sterile distilled water supplemented with 0.01% (v/v) Silwet L-77^13^. Then, the plants were covered with plastic bags for 72 h to maintain relative humidity close to 100%. A positive control for infection was prepared by treating plants only with the *Xv* suspension supplemented with 0.01% Silwet L-77. The negative control consisted of healthy plants dipped in sterile water supplemented with 0.01% Silwet L-77. Five to seven days post-inoculation with *Xv*, the third leaf of each plant (leaf one being the first above the cotyledons) was used to determine bacterial viability. For this, serial dilutions were plated on PYM medium and the CFU per square centimeter of leaf surface were recorded. For the entire duration of the experiment, the plants were kept at 25 °C under a 16 h artificial light/8 dark regime and watered daily with tap water. The assay was repeated three times, with eight plants per treatment.

Stomatal aperture was examined as previously described^47^. Epidermal abaxial peels from leaves of 4-week-old tomato plants were floated in 10:10 buffer (10 mM KCl and 10 mM MES-KOH, pH 6.15) under light for 2.5 h. Then, 20 µM of ABA or 5 µL of CFS-AP1, Met.Ex-AP1, TSB or methanol were added to the medium, and the peels were incubated for another hour. The aperture of 40 stomata were then measured for each treatment at 400× . The data are presented as the average of 120 aperture measurements, collected from three independent experiments.

Callose deposition was analysed as previously described^42^ in *A. thaliana* seedlings, and with some modifications in tomato seedlings. Briefly, 10-day-old *A. thaliana* and tomato seedlings were respectively grown in 12-well and 6-well plates containing liquid MS (Table S5) and treated with 10% CFS-AP1 or Met.Ex-AP1. As a positive control for callose deposition, seedlings were treated with 1 µM flg22. The negative controls consisted of TSB and methanol treatments. After incubation at 22 °C (*A. thaliana*) or 25 °C (tomato) for 24 h, samples were fixed in a 3:1 ethanol/acetic acid mixture for several hours. The fixative solution was renewed several times to ensure both thorough fixation and tissue clearing. The seedlings were rehydrated in 70% ethanol for 2 h, 50% ethanol for an additional 2 h, and water overnight. After two or three washes with water, they were treated with 10% NaOH and incubated at 37 °C for 1 to 2 h to further clear the tissues. They were subsequently washed again with water three or four times and incubated for several hours in 150 mM of K_2_HPO_4_, pH 9.5, with 0.01% aniline blue (Sigma-Aldrich). Finally, they were mounted on slides and observed under a Nikon Eclipse E600 epifluorescence microscope (Nikon, Tokyo, Japan) (excitation 390 nm, emission 460 nm). Callose showed up as bright dots. The images of *A. thaliana* seedlings were captured at 40× and 100× magnification, while those of tomato seedlings were taken at 40× magnification.

### Statistical analysis

All the experiments were repeated three times. For multiple comparisons, analysis of variance (ANOVA) was performed followed by Tukey’s test. Treatments were compared pairwise with Student’s *t*-test. Differences were considered statistically significant at *P* < 0.05. All the data were statistically analysed on GraphPad Prism (version 5.03), and the results were expressed as the mean ± standard deviations (SD).

### Use of artificial intelligence

DeepL Translator and ChatGPT (OpenAI) were used as an aid in drafting the initial version of the manuscript in English. The output was critically reviewed by all the authors, and the final version was extensively revised by a human proofreader.

## Supplementary Information

Supplementary Information.

## Data Availability

The genome assembly and raw sequencing reads are available at NCBI under BioProject PRJNA758981. All other data supporting the findings reported here are available within the article and its supplementary files, or from the corresponding author upon reasonable request.
