# Supplementary material for: Functional characterization of Pseudomonas soli VMAP1 as a biocontrol agent against Xanthomonas vesicatoria in tomato plants
Source: Sci Rep. 2026 Mar 30;16:10586. doi: 10.1038/s41598-026-45489-y (PMC13039529; doi:10.1038/s41598-026-45489-y)
Supplement: Supplementary file 1 — Supplementary Information. [file 41598_2026_45489_MOESM1_ESM.pdf]

SUPPLEMENTARY INFORMATION

Functional characterization of *Pseudomonas soli* VMAP1 as a biocontrol agent against *Xanthomonas vesicatoria* in tomato plants

Tadeo Elías Galván, Valeria Paola Conforte, João Carlos Setubal, Gabriela Petroselli, Rosa Erra-Balsells, Laila Toum, Natalia Mielnichuk, Florencia Malamud, Federico Coluccio Lescow, Florencia Denisse Navarro, Adrián Alberto Vojnov, Pablo Marcelo Yaryura, María Isabel Bianco

Supplementary Table S1: General genomic features of the VMAP1 genome

Feature	Value
Coding sequences (CDSs)	5,060
Functional CDSs	5,011
Pseudogenes	49
RNA genes	80
rRNAs (5S, 16S, 23S)	6, 2, 1
tRNAs	67
NcRNAs	4

Supplementary Figure S1: Clusters of Orthologous Groups (COG) functional classification of VMAP1 CDSs. The figure shows the distribution of COG categories among the CDSs of VMAP1, highlighting metabolic versatility and adaptability.

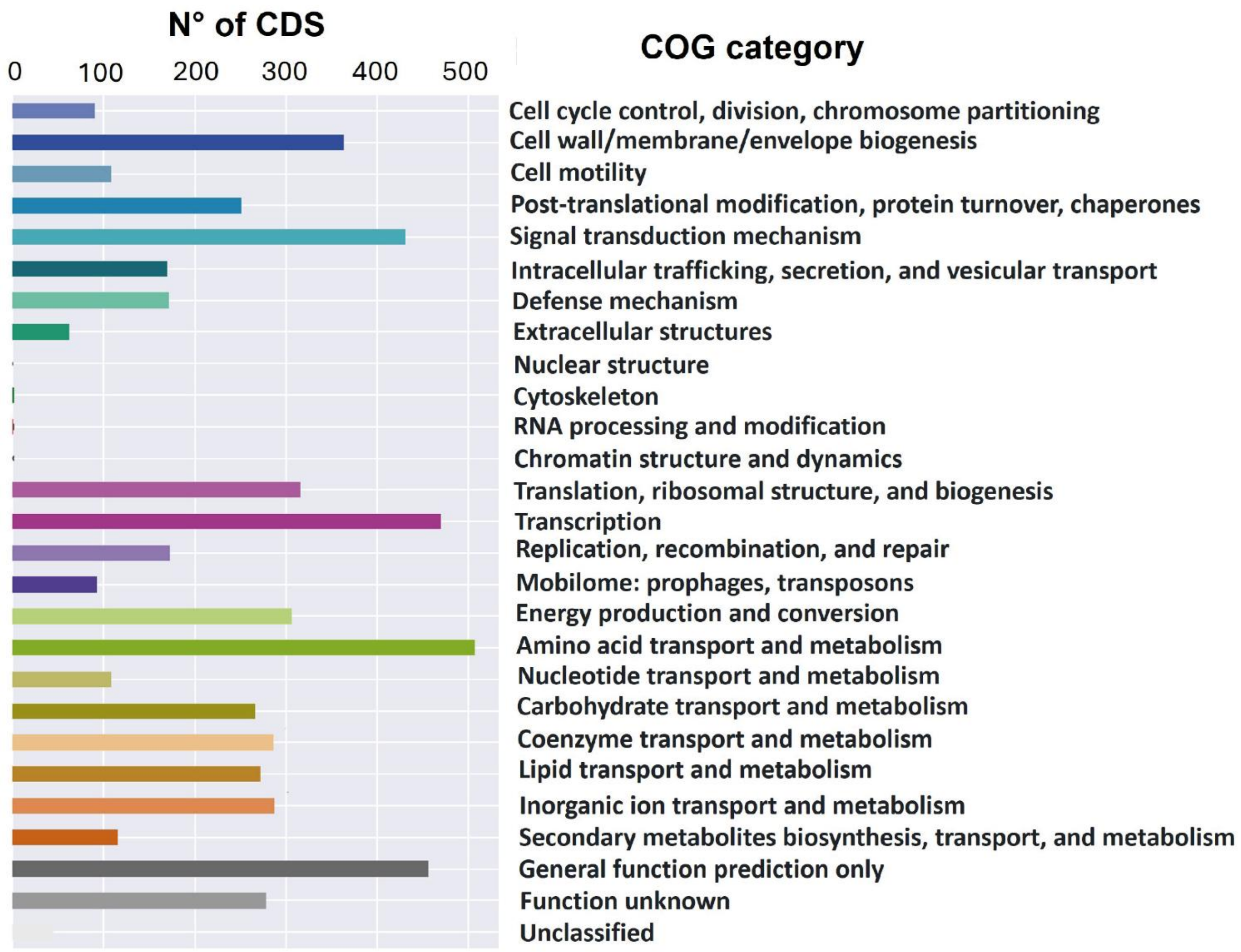

Supplementary Table S2: Biosynthetic and functional gene clusters absent in the genome of VMAP1.

Cluster Type	Cluster Name	Function
NRPS-CLPs	Viscosine, putisolvins, entolysin, arthrofactin, orfamides, CLP8, CLP13, massetolide, jessenipeptin, tolaasin F, bananamide D–G, pseudodesmin, WLIP, syringafactin, cicofactin, asplenin, amphisin, anphisilin.	Biosurfactant<br>Antimicrobial agents
Polyketides	2,4-DAPG, fluoroglucinol	Antibiotics<br>ISR-related metabolites
	Rhizoxins, mupirocin	Antibiotics
	7-hydroxy-tropolone, Sulfazecin, brabantamide	Antimicrobial compounds
Fenazines	PCA, PCN, 2-OH-PCA, 2-OH-Phz, Pyocyanin	Antibiotics<br>ISR-related metabolites
Hydrolytic enzymes	Amylase, ligninase, endglucanases	Carbohydrate degradation
Phytohormone modulation	ACC deaminase<br>IAA biosynthesis proteins<br>Proteins involved in biosynthesis of auxins and phytohormones (cytokinins and gibberellins)<br>Proteins involved in catabolism of auxins and phytohormones<br>Proteins involved in catabolism of phenylacetic acid (PAA)	Modulation of plant development
Siderophores	Pyoverdine, pyochelin, enantio-pyochelin, quinolobactin, tioquinolobactin, acromobactin, hemophore, pseudomonin	Iron acquisition<br>Microbial competition
Exopolysaccharides	Pel, Psl, Cellulose, PNAG, Pea, Peb, Levan	Structural EPSs associated with mature biofilms
Denitrification	NirT, Nor, Nos, Dnr, NirQ	Nitric oxide and N_2O reduction pathways
Resistance system	ArsH, ArsM, ArsI, ArsP	Arsenic metabolism.<br>Arsenic-mediated detoxification
Linear polymer	Oomycin A	Antifungal
Phosphonate	Fosfomycin	Broad-spectrum antibiotic
VOCs	Enzymes involved in the metabolism of 2,3-butanediol.<br>Enzymes involved in the metabolism of acetoin	Plant growth promotion<br>ISR-related metabolites

Supplementary Table S3: Components of the type III secretion system (T3SS) searched in the VMAP1 genome compared with orthologues from *Pseudomonas* spp.

Structural and regulatory components of the T3SS	Sequence identity*
SctD	< 80%
SctE	≥ 80%
SctB	< 80%
SctF	< 80%
SctC	≥ 80%
SctI	≥ 80%
SctJ	≥ 80%
SctK	≥ 80%
SctR	≥ 80%
SctS	≥ 80%
SctT	≥ 80%
SctU	≥ 80%
SctW	Not found
SctQ	≥ 80%
SctV	≥ 80%
SctO	< 80%
SctN	≥ 80%
PscB	< 80%
PscE/EscE/YscE/SsaE family type III secretion system needle protein co-chaperone	< 80%
PscH	Not found
type III export protein PscK	< 80%
PscG	< 80%
Pcr1	< 80%
Pcr2	< 80%
type III secretion system protein Pcr3/PscX	< 80%
type III secretion system chaperone Pcr4/PscY, partial	< 80%
Pcr4	< 80%
PcrR	Not found
PcrG	Not found
SycD/LcrH/PcrH family type III secretion system chaperone	≥ 80%
PcrV	< 80%
PopB	< 80%
PopD	Not found
Exoenzyme S synthesis regulatory protein ExsA	≥ 80%
ExsC	Not found
ExsD	Not found
ExsE	Not found

*CDSs from VMAP1 compared with genes from other *Pseudomonas* spp. Both sequence identity and coverage thresholds were set at 80%. Sequences falling below either threshold were considered absent or divergent.

Supplementary Figure S2: Antibiotic susceptibility profile of VMAP1. Representative images of inhibition zones around antibiotic-impregnated discs on Müller-Hinton agar (MHA) and Trypticase Soy Agar (TSA) plates. Numbers next to the antibiotic names represent their concentration in μg mL^{-1}.

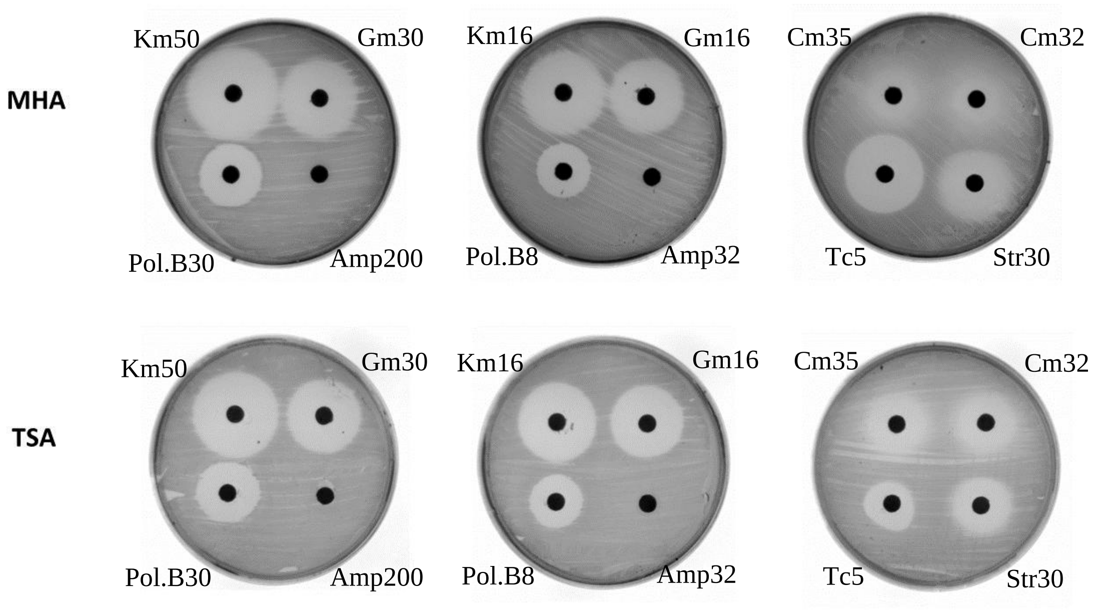

VMAP1 showed resistance to ampicillin and chloramphenicol, and sensitivity to polymyxin B, tetracycline and aminoglycosides (Fig. S2). For these assays, we tested different antibiotic concentrations on TSA and MHA plates: ampicillin (Amp): 8, 32, 200 μg mL^{-1}; chloramphenicol (Cm): 8, 32, 35 μg mL^{-1}; polymyxin B (Pol.B): 2-30 μg mL^{-1}; tetracycline (Tc): 2.5-10 μg mL^{-1}; kanamycin (Km): 4-50 μg mL^{-1}; gentamicin (Gm): 4-30 μg mL^{-1}; streptomycin (Str): 4-30 μg mL^{-1}. The assay is described in the Methods section in the main text.

Supplementary Figure S3: Hydrogen cyanide (HCN) production. Colour change in picrate-soaked filter paper from yellow to brown indicates HCN production by VMAP1.

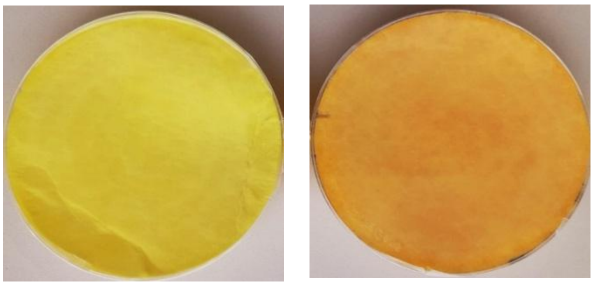

Negative Control **HCN**

Supplementary Table S4: Diagnostic fragments of xantholysin variants detected by MS/MS. Assignment of the main peaks in the ESI-MS/MS spectra of xantholysin A, B, and C, observed as $[M+H]^+$, according to Uchiyama and colleagues[1].

Fragment	Xantholysin A (m/z 1776.09)	Xantholysin B (m/z 1762.07)	Xantholysin C (m/z 1802.10)
y_2	242.15	228.13	242.15
y_3	355.23	341.22	355.23
y_5	596.37	-	-
y_8	895.56	881.54	895.56
y_9	1023.62	1009.60	1023.61
y_{10}	1136.70	-	1136.70
y_{11}	1235.76	-	-
b_1	284.22	284.22	310.24
b_2	413.26	413.26	439.28
b_3	541.32	541.32	567.34
b_4	640.39	640.39	666.41
b_5	753.48	753.47	779.49
b_6	-	881.54	-

Supplementary Figure S4: ESI MS/MS of precursor ion of 1776.08 produced by a methanolic extract (Met.Ex-AP1) obtained from CFS-AP1 in positive ion mode identified as **xantholysin A** (see Table S4 for details).

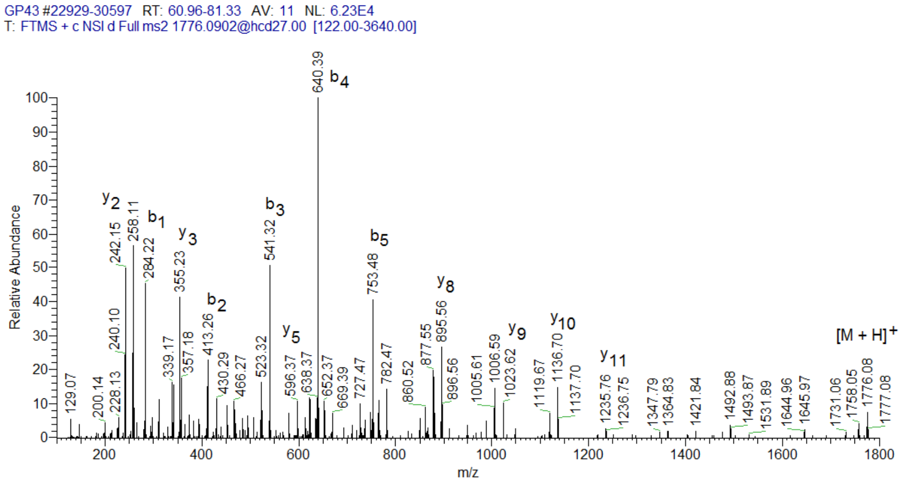

Supplementary Figure S5: ESI MS/MS of precursor ion of 1762.07 produced by a methanolic extract (Met.Ex-AP1) obtained from CFS-AP1 in positive ion mode identified as **xantholysin B** (see Table S4 for details).

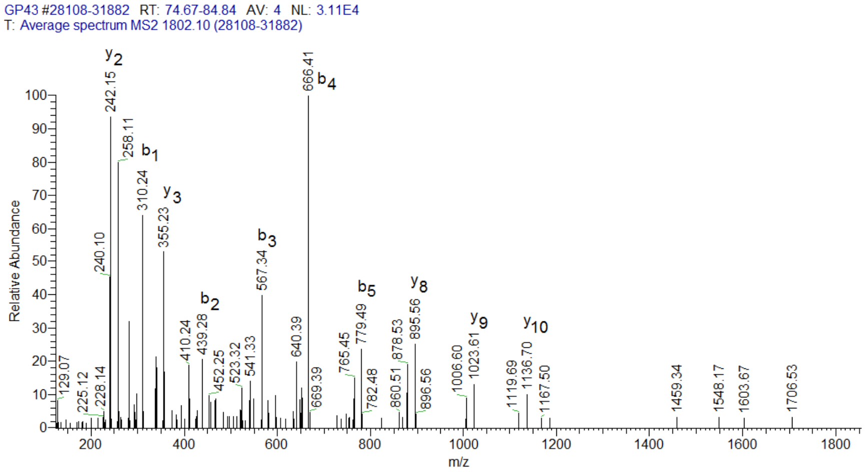

Supplementary Figure S6: ESI MS/MS of precursor ion of 1802.10 produced by a methanolic extract (Met.Ex-AP1) obtained from CFS-AP1 in positive ion mode identified as **xantholysin C** (see Table S4 for details).

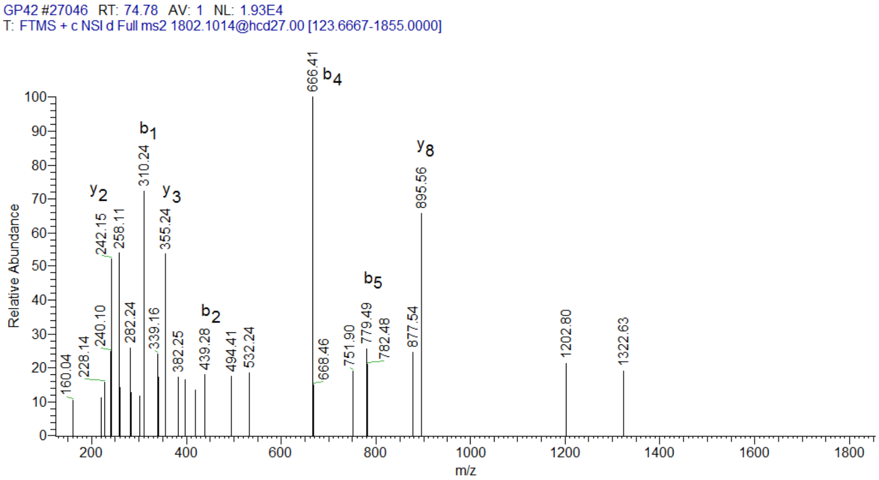

Supplementary Table S5: Culture media used in this study

Medium	Abbreviation	Reference
Trypticase Soy Broth	TSB	[2]
Trypticase Soy Agar	TSA	[2]
King’s B medium	KB	[3]
Luria-Bertani medium	LB	[4]
Mueller-Hinton agar	MHA	[5]
PYM medium	-	[6]
M9 minimal medium	M9	[7]
Yeast minimal medium	YMM	[8]
Davis minimal medium	DMM	[9]
Murashige and Skoog medium	MS	[10]
Nutrient Yeast Glycerol Broth	NYGB	[11]

References

1. Uchiyama, C. *et al.* Structural revision of natural cyclic depsipeptide MA026 established by total synthesis and biosynthetic gene cluster analysis. **Angew. Chem. Int. Ed.** (2021). https://doi.org/10.1002/anie.202015193
2. Zimbro, M. J. & Power, D. A. *Difco & BBL manual: manual of microbiological culture media.* (Becton Dickinson and Co., Sparks, 2009).
3. Nichols, D., Chmiel, J. & Berger, M. Chronic inflammation in the cystic fibrosis lung: alterations in inter- and intracellular signaling. **Clin. Rev. Allergy Immunol.** 34, 146–162 (2008).
4. Molina-Santiago, C. *et al.* Efflux pump-deficient mutants as a platform to search for microbes that produce antibiotics. **Microb. Biotechnol.** (2015). https://doi.org/10.1111/1751-7915.12295
5. Mueller, J. H. & Hinton, J. A protein-free medium for primary isolation of the gonococcus and meningococcus. **Proc. Soc. Exp. Biol. Med.** (1941). https://doi.org/10.3181/00379727-48-13311
6. Cadmus, M. C. *et al.* Colonial variation in *Xanthomonas campestris* NRRL B-1459 and characterization of the polysaccharide from a variant strain. **Can. J. Microbiol.** 22, 942–948 (1976).
7. Sambrook, J. & Russell, D. W. *Molecular cloning: a laboratory manual.* (Cold Spring Harbor Laboratory Press, Cold Spring Harbor, 2001).
8. Sherwood, R. T. & Huisingh, D. Calcium nutrition and resistance of alfalfa to *Ditylenchus dipsaci*. **J. Nematol.** 2, 316–323 (1970).
9. Davis, B. D. & Mingioli, E. S. Mutants of *Escherichia coli* requiring methionine or vitamin B12. **J. Bacteriol.** (1950). https://doi.org/10.1128/jb.60.1.17-28.1950
10. Murashige, T. & Skoog, F. A revised medium for rapid growth and bio assays with tobacco tissue cultures. **Physiol. Plant.** (1962). https://doi.org/10.1111/j.1399-3054.1962.tb08052.x
11. Daniels, M. J., Barber, C. E., Turner, P. C., Cleary, W. G. & Sawczyc, M. K. Isolation of mutants of *Xanthomonas campestris* pathovar *campestris* showing altered pathogenicity. **J. Gen. Microbiol.** 129, 3095–3108 (1983). https://doi.org/10.1099/00221287-129-10-3095
